# Historical Contingency Drives Freshwater Microbial Community Assembly Across Successional Time

**DOI:** 10.1002/ece3.73819

**Published:** 2026-06-09

**Authors:** Fenguo Zhang, Xiaoting Zhang, Dongqing Yan, Yufeng Jing, Yongji Wang

**Affiliations:** ^1^ College of Life Science, Shanxi Engineering Research Center of Microbial Application Technologies Shanxi Normal University Taiyuan Shanxi China

**Keywords:** community assembly, dispersal limitation, environmental selection, historical contingency, priority effects

## Abstract

Microbial community assembly is governed by the interplay among historical contingency, environmental selection, and dispersal, yet their relative importance and temporal dynamics remain poorly resolved, particularly in freshwater ecosystems. Most previous studies have examined these drivers in isolation, limiting our ability to predict microbial community trajectories under changing environmental and dispersal regimes. Here, we conducted a 60‐day full‐factorial reciprocal transplant microcosm experiment using freshwater bacterial communities, manipulating historical source communities, environmental media, and the intensity of external immigration (ambient vs. enhanced). Bacterial community dynamics were tracked at early (day 13) and late (day 60) successional stages using 16S rRNA gene amplicon sequencing. We found that relative dispersal limitation strongly influenced community assembly during early succession, with higher immigration rates increasing alpha diversity (e.g., ASV richness *F* = 23.45, *p* < 0.001) and altering community composition. However, this effect weakened over time, indicating a transition toward dispersal saturation. In contrast, the influence of historical contingency persisted throughout the experiment and became the strongest single driver of community composition at the late successional stage (*R* ^2^ = 13.34%), exceeding the explanatory power of environmental selection (*R* ^2^ = 5.02%). Communities sharing the same historical source consistently followed distinct assembly trajectories, regardless of environmental medium or immigration rate. Together, our results demonstrate a time‐dependent shift in the mechanisms governing freshwater microbial community assembly, from early relative dispersal limitation to late‐stage historical dominance driven by priority effects. These findings highlight the critical and lasting role of historical contingency in shaping microbial community structure and suggest that community assembly is governed by a dynamic interplay of mechanisms, with historical contingency playing a greater, lasting role that leads to strong path dependency.

## Graphical abstract

Using a 60‐day full‐factorial reciprocal transplant microcosm experiment, we quantified the relative contributions of historical contingency, environmental selection, and dispersal limitation to freshwater bacterial community assembly. We show a pronounced temporal shift from early dispersal‐driven assembly to late‐stage dominance of historical contingency via priority effects. These results reveal strong path dependency and predictable assembly trajectories in freshwater microbial communities under environmental change.

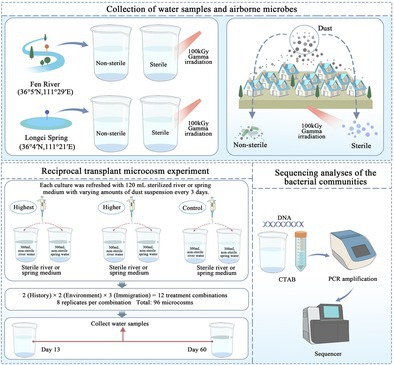

## 1 Introduction

Microorganisms are fundamental components of freshwater ecosystems, where they regulate organic matter turnover, nutrient cycling, and overall ecosystem functioning (Battin et al. 2016; Falkowski et al. 2008). Consequently, the assembly of freshwater microbial communities has attracted increasing interest, particularly in the context of environmental change, biodiversity loss, and ecosystem restoration (Hester et al. 2021; Savio et al. 2015). However, despite extensive descriptive studies documenting spatial and temporal patterns of microbial diversity, the mechanisms governing freshwater microbial community assembly remain incompletely understood (Liu et al. 2015; Logue et al. 2011).

Ecological theory recognizes that community assembly is jointly shaped by deterministic processes, such as environmental selection, and stochastic processes, including dispersal limitation and ecological drift (Vellend 2010; Zhou and Ning 2017). In microbial ecology, this framework has been widely applied to explain variation in community composition across environmental gradients and spatial scales (Hanson et al. 2012; Stegen et al. 2013). More recently, historical contingency—the influence of initial community composition on subsequent assembly trajectories—has emerged as a critical yet underexplored component linking deterministic and stochastic processes (Fukami 2015; Nemergut et al. 2013). Through priority effects, early colonizing taxa can preempt resources, modify local environments, and establish interaction networks that constrain the establishment success of later arrivals, potentially leading to long‐lasting divergence among communities exposed to similar contemporary conditions (Chase 2007; Fukami and Morin 2003).

Although the importance of history, environment, and dispersal has been recognized conceptually, their relative contributions and temporal dynamics remain unresolved, particularly in freshwater ecosystems (Lindström and Langenheder 2012; Székely and Langenheder 2014). Freshwater ecosystems, such as rivers and springs, are particularly well‐suited for examining this interplay. They are characterized by discrete habitat patches embedded within a terrestrial matrix, creating natural metacommunities with defined boundaries. They experience strong, often fluctuating environmental gradients (e.g., temperature, nutrients, flow) that impose deterministic selection. Simultaneously, they are open to dispersal from upstream, groundwater, and aerial sources, making dispersal a key variable. Furthermore, their relatively simple physical structure compared to soils allows for controlled manipulation in microcosms, enabling the direct, experimental disentanglement of assembly drivers that is often difficult in more complex systems. Most existing studies focus on single drivers or pairwise interactions and rely heavily on field observations, making it difficult to disentangle causal relationships among assembly processes (Dini‐Andreote et al. 2015; Evans et al. 2017). As a result, it remains unclear whether historical effects persist or decay over time, how dispersal limitation changes across successional stages, and under what conditions historical contingency can outweigh environmental filtering to shape long‐term microbial community structure (Grainger et al. 2019; Karimi et al. 2017; Peay et al. 2016; Vass and Langenheder 2017).

**FIGURE 1:**
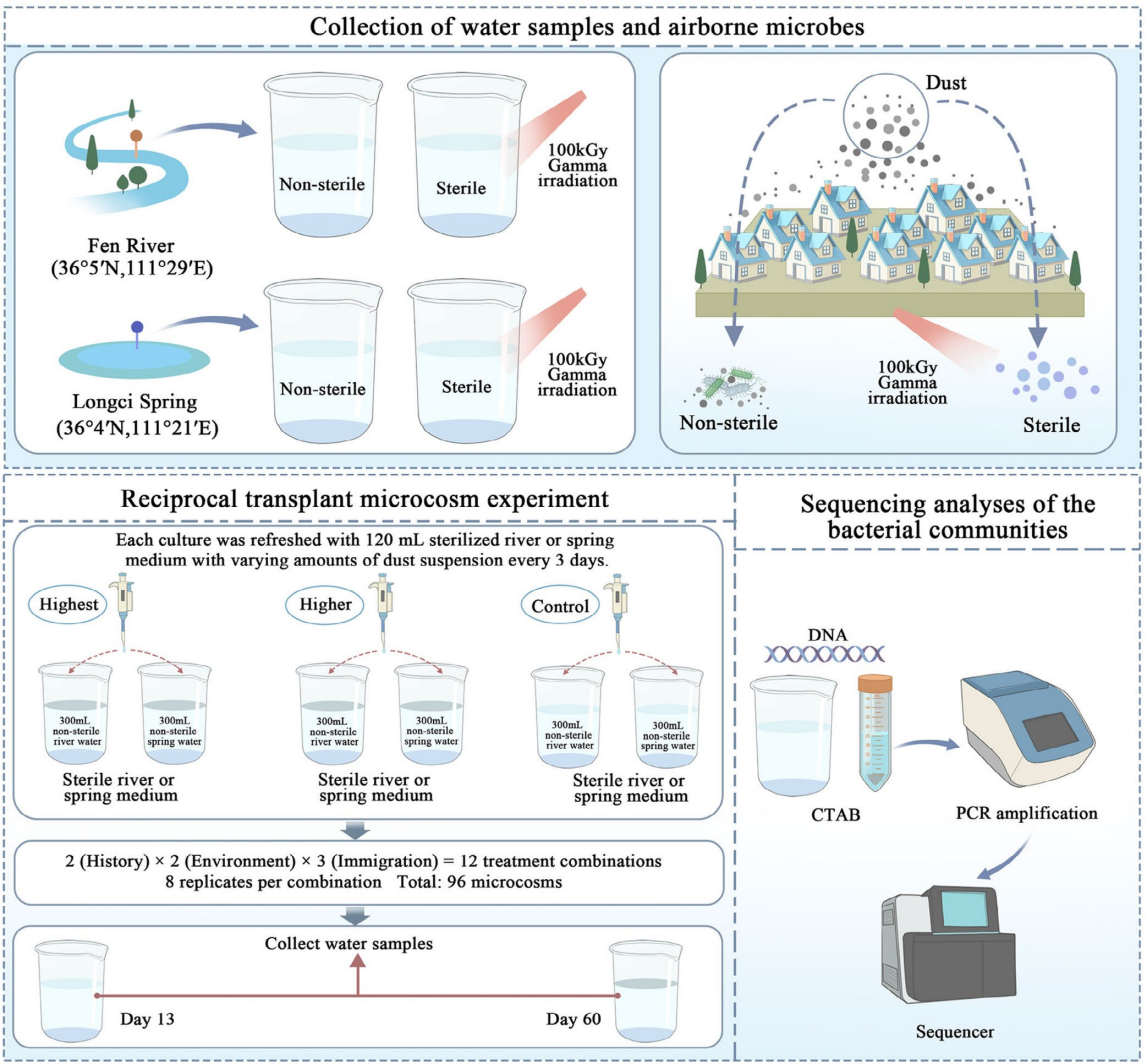
Experimental design of the reciprocal transplant microcosm study.

To address these gaps, we conducted a controlled, full‐factorial reciprocal transplant microcosm experiment to disentangle the relative and interactive effects of historical contingency, environmental selection, and relative dispersal limitation on freshwater bacterial community assembly (Figure 1). We established microcosms using non‐sterilized source communities from a river and a spring ecosystem, incubated them in sterilized river or spring water media, and manipulated immigration rates to simulate different dispersal intensities (Albright and Martiny 2018; Zhang et al. 2019). Community dynamics were monitored at an early (day 13) and a late (day 60) successional stage, allowing us to explicitly assess the temporal shifts in assembly mechanisms (Ferrenberg et al. 2013).

Specifically, we aimed to: (i) quantify the independent and interactive contributions of historical source, environmental medium, and dispersal to bacterial community composition; (ii) determine whether the strength of relative dispersal limitation changes across successional stages; and (iii) evaluate whether historical contingency can persist or even outweigh environmental selection during late‐stage community assembly. Based on current community assembly theory, we hypothesized that environmental selection would exert a strong influence throughout the experiment but weaken over time, relative dispersal limitation would dominate early assembly but diminish as communities approached saturation (Cadotte 2006; Mittelbach and Schemske 2015), and historical contingency would persist and increasingly shape long‐term community trajectories through priority effects (Chase and Leibold 2003; Fukami and Morin 2003).

## 2 Materials and Methods

### 2.1 Collection of Airborne Microbes

Airborne microbes were collected from undisturbed outdoor surfaces at 10 sites within 20 km of the experimental location in June 2020 (Table S1). Dust samples from all sites were equally pooled to create a “regional air pool” (Choudoir et al. 2018). A portion of this homogenized dust was sterilized by 100 kGy gamma irradiation for subsequent experiments. The community structure of bacteria in dust was analyzed by sequencing.

### 2.2 Collection of Water Samples

Water samples were collected from the Fen River and Longci Spring in June 2020. After filtration, a portion of the water was sterilized (100 kGy gamma irradiation) to serve as sterile medium. Although high‐dose gamma irradiation may alter certain chemical properties of the water, it provides an effective approach to eliminate biological activity while maintaining a consistent environmental template across treatments. The sterility of irradiated samples was confirmed by culture assays and the absence of amplifiable 16S rRNA gene (Zhang and Zhang 2015, 2016). The physicochemical properties and initial bacterial community structure of the water were provided (Tables S2 and S3). The two water sources were chosen for their distinct physicochemical profiles to represent different environmental templates.

Microcosm experiments were conducted outdoors within a defined 3 m × 3 m area during July and August 2020. To control for potential microclimatic variation within the outdoor experimental area (e.g., shading, wind patterns), the 96 microcosms were arranged in a randomized complete block design. The 3 m × 3 m area was divided into eight spatial blocks, and one complete set of all 12 treatment combinations was randomly placed within each block. Microcosms were re‐randomized within their blocks weekly to minimize persistent positional effects.

### 2.3 Reciprocal Transplant Microcosm Experiment

Every initial microcosm contained non‐sterile water from one sampling site. Non‐sterilized river water and spring water were inoculated into glass beakers (300 mL of water in 500 mL sterile beakers). The microcosms were open to the air (lids removed). Each culture was refreshed with 120 mL of sterile medium every 3 days. Microcosms initiated with the Fen River community were refreshed with either sterilized Fen River or Longci Spring medium, and the same protocol was applied to microcosms initiated with the Longci Spring community.

One elevated immigration (highest immigration) treatment was applied to one‐third of the microcosms: at each medium refreshment, each microcosm received 1 mL of dust microbial suspension. A second elevated immigration (higher immigration) treatment was applied to another one‐third: at each transfer, each microcosm received a mixture of 0.1 mL dust suspension and 0.9 mL sterilized suspension. The ambient immigration (control) treatment microcosms received 1 mL of sterilized dust suspension at each transfer.

The reciprocal transplant experiment followed a full factorial design with three variables: history source community (non‐sterilized Fen River vs. Longci Spring), environment medium (sterilized Fen River vs. Longci Spring), and the intensity of external immigration (ambient vs. enhanced). We maintained eight replicates for each treatment combination, resulting in a total of 96 microcosms (Table S4). The microcosms of non‐sterilized Fen River history, sterilized Fen River environment were referred to as “Fen‐Fen” in the text. The microcosms of non‐sterilized Longci Spring history, sterilized Longci Spring environment were referred to as “Long‐Long”. The microcosms of non‐sterilized Fen River history, sterilized Longci Spring environment were referred to as “Fen‐Long”. The microcosms of non‐sterilized Longci Spring history, sterilized Fen River environment were referred to as “Long‐Fen”. Samples were collected on day 13 (after 4 transfers) and day 60 (after 20 transfers) for bacterial community structure analysis via sequencing.

### 2.4 Bacterial Community Sequencing Analysis

Bacterial community analysis was conducted via 16S rRNA gene amplicon sequencing of the V4 hypervariable region. Total DNA was extracted from water (Fen River, Longci Spring, microcosms) and dust samples using the CTAB method. The V4 region was amplified using barcoded universal primers 515F and 806R (Caporaso et al. 2011). PCR products were purified, verified, and pooled for sequencing. Paired‐end sequencing (2 × 250 bp) was performed on an Illumina NovaSeq 6000 platform (Novogene Technology Co. Ltd., Tianjin, China). Bioinformatic processing included merging reads (FLASH) (Magoč and Salzberg 2011), quality filtering (fastp) (Bokulich et al. 2013), and chimera removal (UCHIME vs. Silva 138.1) (Edgar et al. 2011). Amplicon Sequence Variants (ASVs) were generated by denoising with DADA2 in QIIME2 (Callahan et al. 2016). Taxonomic annotation was assigned using a Naïve Bayes classifier (QIIME2) against the Silva 138.1 database.

We acknowledge that this approach deviates from the standard DADA2 workflow, and therefore our results should be interpreted with caution regarding fine‐scale sequence error modeling. However, since all samples were processed using the same pipeline, relative comparisons among treatments remain valid.

### 2.5 Data Analysis

#### 2.5.1 Alpha and Beta Diversity Analysis

Bacterial community analyses were performed in R using the vegan package. Alpha diversity (ASV richness, Shannon index, Faith's PD) was calculated from rarefied ASV tables (40,449 sequences/sample) (Bray and Curtis 1957; Oksanen et al. 2020).

Beta diversity was assessed using Bray–Curtis dissimilarity matrices for all samples (*n* = 96 across treatments and time points). To quantify the influence of history, selection, and relative dispersal limitation, the proportion of variance explained by each factor and their interactions was assessed via PERMANOVA (adonis, 9999 permutations) on the Bray‐Curtis matrix. Community patterns were visualized using NMDS based on the same Bray–Curtis dissimilarity (Anderson 2001; Zhang et al. 2019).

#### 2.5.2 Phylogenetic and Taxonomic Composition Analysis

To assess the phylogenetic structure and taxonomic composition of bacterial communities across treatments, we performed weighted UniFrac distance analysis and phylum‐level relative abundance analysis. Weighted UniFrac distances were calculated based on the phylogenetic tree constructed from the ASV sequences, incorporating both presence/absence and abundance information (Lozupone and Knight 2005). Hierarchical clustering (UPGMA) was performed on the weighted UniFrac distance matrix to visualize the phylogenetic similarity among microcosms. For phylogenetic analyses (weighted UniFrac), a multiple sequence alignment of the representative ASV sequences was first generated using MAFFT (version 7.5). The alignment was then used to construct a rooted phylogenetic tree with FastTree (version 2.1), which infers approximately maximum‐likelihood phylogenetic trees from alignments of nucleotide sequences. Additionally, the relative abundance of major bacterial phyla was summarized from the taxonomic annotation of ASVs and visualized in a stacked bar plot. These analyses were conducted in QIIME2 and R using the phyloseq package (McMurdie and Holmes 2013).

## 3 Results

### 3.1 Alpha Diversity Reveals Temporal Shifts in Relative Dispersal Limitation

On day 13, in the Fen‐Fen microcosms, ASV richness significantly increased with immigration (*p* < 0.001), while no significant change was observed in the Long‐Long microcosms (*p* = 0.070). The Shannon index significantly increased in both homogenous treatments (both *p* < 0.001). Faith's PD showed no significant change in either treatment (both *p* > 0.05).

By day 60, ASV richness exhibited a decreasing trend with immigration in both homogenous treatments, reaching significance only in Long‐Long (*p* = 0.002) but not in Fen‐Fen (*p* = 0.151). The Shannon index significantly increased in Fen‐Fen (*p* < 0.001) but decreased in Long‐Long (*p* < 0.001). Faith's PD significantly decreased in both treatments (both *p* < 0.05).

In the cross‐transplant treatments, on day 13, both ASV richness and Shannon index significantly increased in Fen‐Long and Long‐Fen microcosms (richness: *p* < 0.01; Shannon: *p* < 0.001). Faith's PD significantly increased only in Long‐Fen (*p* = 0.004).

By day 60, only ASV richness in Long‐Fen showed a significant increase with immigration (*p* = 0.043), while no significant changes were detected in the other alpha diversity metrics for either cross‐transplant treatment (all *p* > 0.05).

Collectively, these patterns indicate that freshwater bacterial community assembly was strongly constrained by relative dispersal limitation during early succession, whereas the influence of dispersal weakened as communities matured, consistent with a transition toward dispersal saturation at later stages.

### 3.2 Community Composition Reflects the Relative Roles of History, Environment, and Dispersal

#### 3.2.1 Historical Contingency and Environmental Selection Jointly Structure Community Composition

To disentangle the relative effects of history, selection, and relative dispersal limitation on community structure, we conducted a series of PERMANOVA analyses based on Bray–Curtis dissimilarity matrices.

First, analyzing the effects of history and environment at fixed immigration levels revealed that historical source, environmental medium, and their interaction all had highly significant effects on community composition variation across all three immigration treatments (control, higher, highest) and at both time points (*p* = 0.001, Table 1). Notably, the independent effect of historical source was most prominent by the experiment's end, explaining the highest proportion of community variation (Day 60, *R* ^2^ = 13.34%). This was substantially higher than the independent effect of environmental selection at the same time point (*R* ^2^ = 5.02%, Table 2). Importantly, these patterns were observed after standardizing sequencing depth across samples, indicating that they are unlikely to be driven solely by differences in initial richness. Specifically, the proportion of variation explained by the environment (*R* ^2^) decreased over time (from day 13 to day 60) within the same immigration treatment. In contrast, the variation explained by historical source tended to increase with higher immigration rates (from control to highest) and maintained a high explanatory power in the highest immigration treatment by the end of the experiment (day 60, *R* ^2^ = 22.15%).

**TABLE 1:** Non‐parametric MANOVA analysis of the Bray–Curtis dissimilarities among experimental communities within each immigration rate in reciprocal transplants experiment.

		History source			Environment			Environment×History			Residuals
		*F*	*p*	*R* ^2^	*F*	*p*	*R* ^2^	*F*	*p*	*R* ^2^	*R* ^2^
Ambient immigration	Day13	18.0495	0.001***	0.28355	9.6532	0.001***	0.15165	7.9528	0.001***	0.12494	0.43987
	Day60	7.3957	0.001**	0.18207	5.0433	0.001***	0.12416	1.938	0.001***	0.10291	0.59085
Higher immigration	Day13	31.189	0.001***	0.34335	16.491	0.001***	0.18154	15.157	0.001***	0.16686	0.30824
	Day60	11.5636	0.001***	0.21165	8.7533	0.001***	0.16021	6.3194	0.001***	0.1156	0.51248
Highest immigration	Day13	21.6167	0.001***	0.32332	7.3812	0.001***	0.1101	9.9858	0.001***	0.14895	0.41764
	Day60	10.3169	0.001***	0.22154	3.5556	0.002**	0.07635	4.6973	0.001***	0.10087	0.60125

Abbreviations: *F*, *F*‐test statistic; *p*, proportion of randomization trials with more extreme values of *F*; *R* ^2^, explainable proportions by certain factor.

** A *p*‐value of less than 0.01 indicates a highly signiffcant difference.

*** A *p*‐value of less than 0.001 indicates an extremely signiffcant difference.

**TABLE 2:** Non‐parametric MANOVA analysis of the Bray–Curtis dissimilarities among experimental communities within three factors in reciprocal transplants experiment.

Source of variance	Day13						Day60					
	Df	SS	MS	*F*	*R* ^2^	*p*	Df	SS	MS	*F*	*R* ^2^	*p*
Environment	1	2.0717	2.0717	25.447	0.09107	0.001***	1	1.4100	1.4100	7.9697	0.05019	0.001***
Immigration	2	4.9293	2.4646	30.274	0.21669	0.001***	2	3.1852	1.5926	9.0020	0.11338	0.001***
History source	1	4.5374	4.5374	55.734	0.19946	0.001***	1	3.7471	3.7471	21.1802	0.13338	0.001***
Environment:Immigration	2	0.6699	0.3349	4.114	0.02945	0.001***	2	1.5763	0.7881	4.4549	0.05611	0.001***
Environment:History	1	2.0012	2.0012	24.581	0.08797	0.001***	1	1.2402	1.2402	7.0103	0.04415	0.001***
Immigration:History source	2	1.0888	0.5444	6.687	0.04768	0.001***	2	1.3681	0.6840	3.8655	0.04870	0.001***
Environment:Immigration:History source	2	0.6116	0.3058	3.756	0.02689	0.001***	2	1.4129	0.7065	3.9932	0.05029	0.001***
Residuals	84	6.8386	0.0814		0.30062		80	14.1532	0.1769		0.50380	
Total	95	22.7483			1.00000		91	28.0930			1.00000	

Abbreviations: Df, degrees of freedom; *F*, *F*‐test statistic; MS, mean squares; *p*, proportion of randomization trials with more extreme values of *F; R* ^*2*^, explainable proportions by certain factor; SS, sums of squares.

*** A *p*‐value of less than 0.001 indicates an extremely signiffcant difference.

Second, a comprehensive three‐factor (Environment, History, Immigration) PERMANOVA model assessed the independent and joint contributions of each driver. The results demonstrated that environmental selection, historical source, and relative dispersal limitation (immigration rate) all independently exerted highly significant effects on community composition on both day 13 and day 60 (*p* < 0.001). Notably, the independent effect of historical source was most prominent by the experiment's end, explaining the highest proportion of community variation (Day 60, *R* ^2^ = 13.34%). Furthermore, all two‐way (Environment × History, Environment × Immigration, History × Immigration) and the three‐way (Environment × History × Immigration) interactions were also highly significant (*p* < 0.001), indicating these assembly processes did not operate independently but exhibited complex synergistic or antagonistic relationships (Table 2).

Together, these results demonstrate that historical source, environmental medium, and dispersal all significantly structured community composition, but the independent effect of historical contingency became strongest at the late successional stage.

#### 3.2.2 Ordination Patterns Reveal Distinct Assembly Trajectories Across Treatments

**FIGURE 2:**
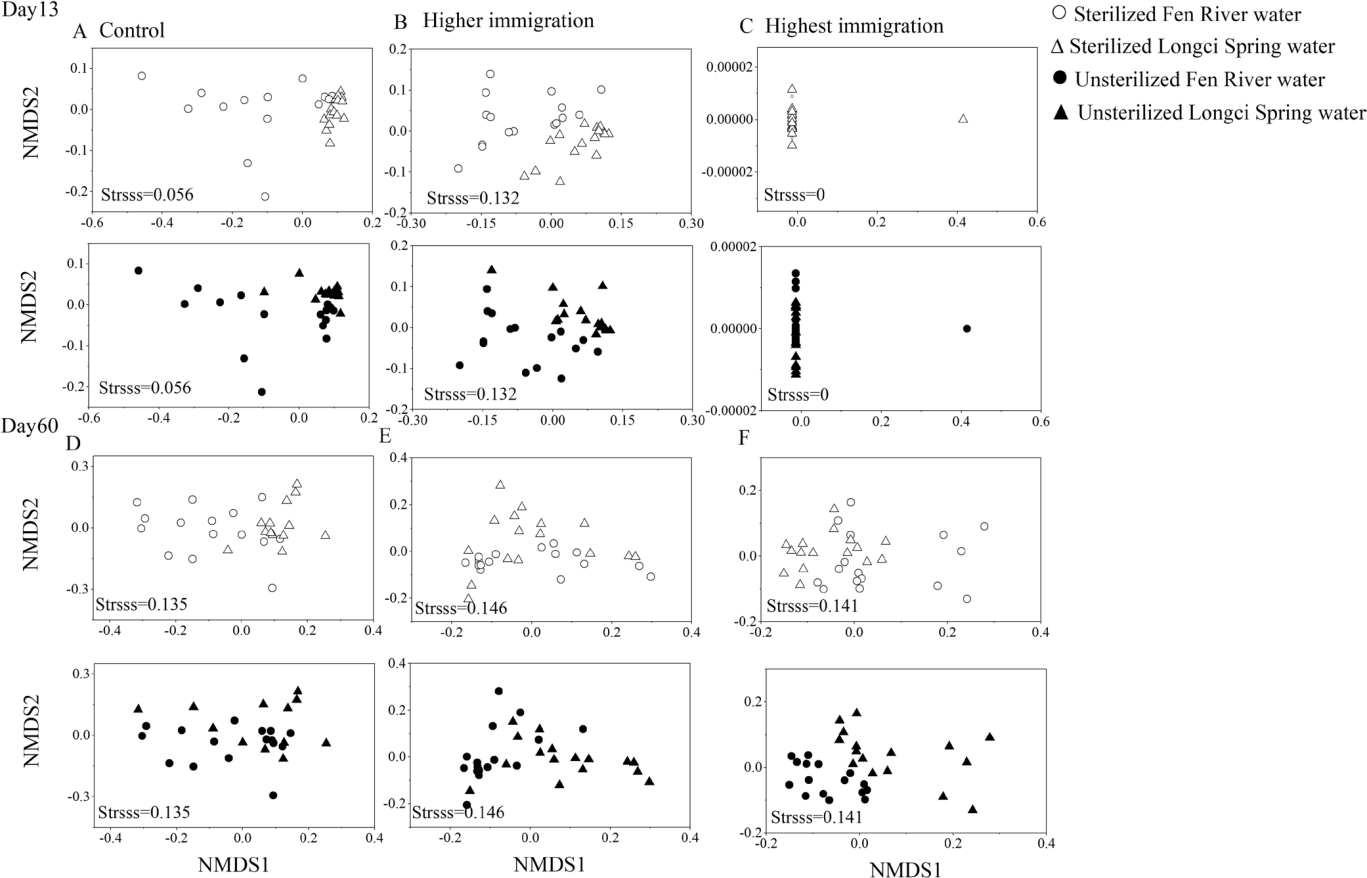
Non‐metric multidimensional scaling ordination of experimental communities on the 13th (A–C) and the 60th (D–F) day for each immigration rate. Each point represents an experimental community, and the distance between two points represents the dissimilarities between communities. Stress is a function assessing how well the derived two‐dimensional plot fits the pairwise dissimilarity matrix (stress > 0.2, poor; stress = 0.1, fair; stress < 0.05, good; stress = 0, perfect). The pairwise dissimilarities of all communities at a specific immigration rate are shown twice; in the upper panel, points having the same shape were communities originating from the same environment (hollow circle represented sterilized Fen River water medium and hollow triangle represented sterilized Longci Spring water medium), and in the lower panel points having the same shape were communities originating from the same history source (solid circle represented unsterilized Fen River water and solid triangle represented unsterilized Longci Spring water).

**FIGURE 3:**
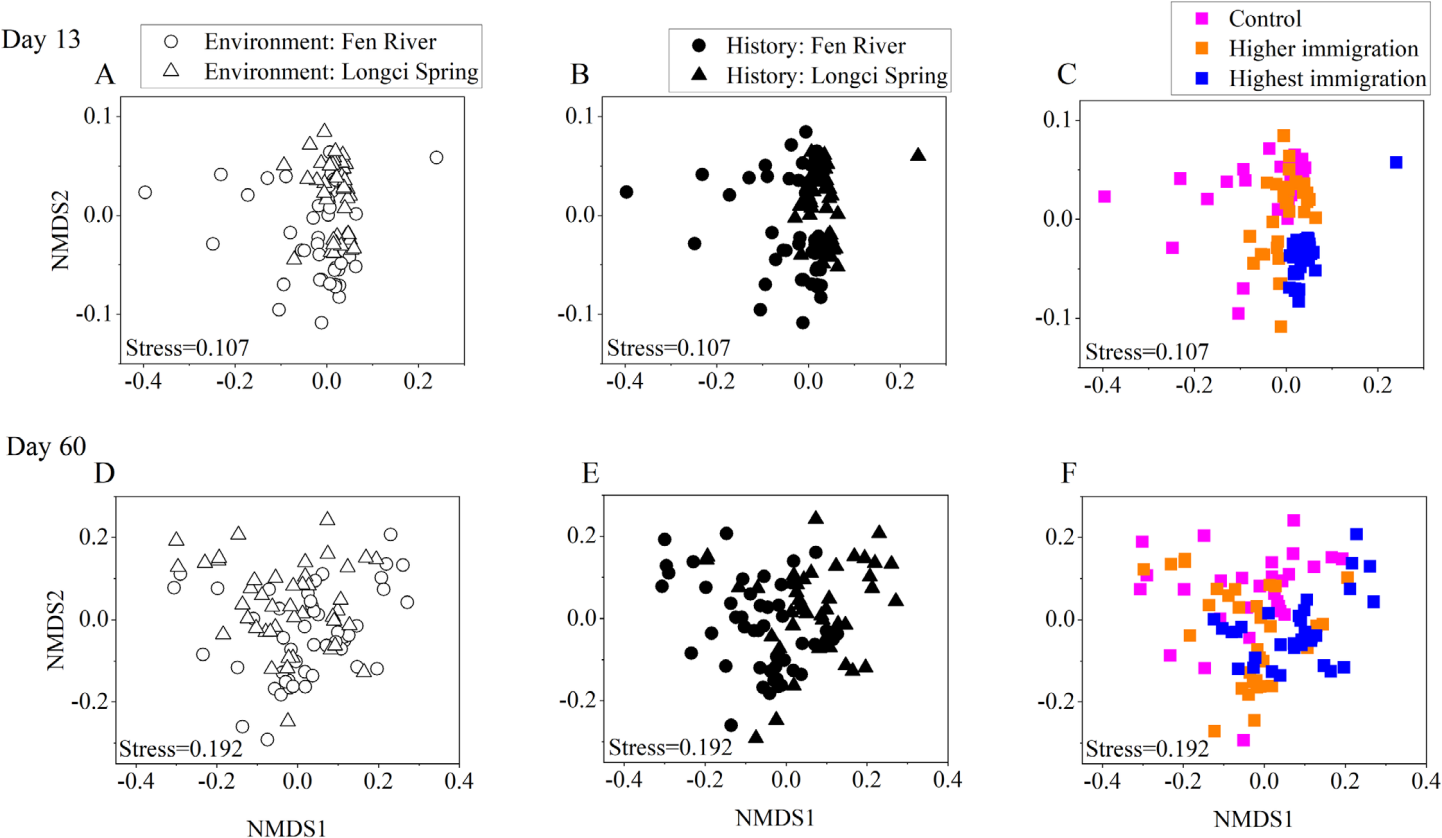
Non‐metric multidimensional scaling ordination of experimental communities on the 13th (A–C) and the 60th (D–F) day for environment, history source and immigration rate treatments. Each point represents an experimental community, and the distance between two points represents the dissimilarities between communities. Stress is a function assessing how well the derived two‐dimensional plot fits the pairwise dissimilarity matrix (stress > 0.2, poor; stress = 0.1, fair; stress < 0.05, good; stress = 0, perfect). The pairwise dissimilarities of all communities at a specific environment or immigration rate are shown; in the figures A and D, points having the same shape were communities originating from the same environment (hollow circle represented sterilized Fen River water medium and hollow triangle represented sterilized Longci Spring water medium); in the figures B and E, points having the same shape were communities from the same history source (solid circle represented unsterilized Fen River water and solid triangle represented unsterilized Longci Spring water); and in the figures C and F, points having the same color were communities originating from the same immigration rate.

Non‐metric multidimensional scaling (NMDS) ordination provided intuitive visual evidence supporting the above statistical results (Figures 2 and 3). The ordination revealed that microcosm communities sharing the same environmental medium or the same historical source consistently exhibited clear clustering trends, both in the early (day 13) and late (day 60) stages. This indicates that community development was consistently strongly influenced by its initial source (history) and the prevailing environment (selection), forming unique assembly trajectories. Moreover, communities under different immigration rates but sharing the same environment and history background also clustered together, further supporting the pattern that dispersal processes interact with local conditions to jointly shape communities.

The consistent clustering of communities by historical source and environmental medium across immigration treatments suggests the emergence of distinct assembly trajectories shaped by interacting assembly processes.

### 3.3 Phylogenetic and Taxonomic Patterns Reinforce Persistent Assembly Constraints

Phylogenetic clustering based on weighted UniFrac distances revealed clear and persistent separation of bacterial communities according to both historical source and environmental medium across successional stages (Figure S1). Microcosms originating from the same historical source (Fen River vs. Longci Spring) or incubated in the same environmental medium consistently clustered together at both early (day 13) and late (day 60) time points, indicating that historical legacy and environmental filtering imposed strong and lasting phylogenetic constraints on community assembly.

Taxonomic composition at the phylum level further supported these phylogenetic patterns. Across all treatments, Proteobacteria dominated the bacterial communities, followed by Bacteroidota, Cyanobacteria, Actinobacteriota, and Firmicutes (Supporting Information). Notably, microcosms with Fen River history exhibited a higher relative abundance of Cyanobacteria, particularly in the Fen‐Fen treatment, whereas communities originating from Longci Spring were characterized by higher proportions of Bacteroidota. Environmental medium also influenced taxonomic structure, with microcosms incubated in sterilized Fen River water showing increased relative abundance of Proteobacteria, while those in sterilized Longci Spring water favored Bacteroidota and Cyanobacteria. These compositional differences were consistent with the physicochemical characteristics of the two water sources.

Together, the concordance between phylogenetic clustering and taxonomic composition demonstrates that historical contingency and environmental selection jointly constrained freshwater bacterial community assembly, reinforcing divergent and persistent assembly trajectories across treatments and over time.

## 4 Discussion

In this study, we experimentally disentangled the relative and interactive effects of historical contingency, environmental selection, and relative dispersal limitation on freshwater bacterial community assembly across successional time (Figure 1). Using a controlled, full‐factorial reciprocal transplant microcosm experiment, we highlight a pronounced temporal shift in the dominant assembly mechanisms. Dispersal limitation played a critical role during early community establishment, when increased immigration enhanced diversity and altered community composition, indicating an open assembly window (De Meester et al. 2016; Lear et al. 2013). As succession progressed, however, the influence of dispersal weakened markedly, while historical contingency persisted and ultimately became the strongest single driver shaping community structure (Chase and Leibold 2003; Vannette and Fukami 2014). By the late stage, communities originating from the same historical source consistently converged on distinct assembly trajectories, regardless of environmental medium or immigration intensity. Together, these results highlight strong path dependency in freshwater microbial community assembly and underscore the lasting influence of historical legacy over successional time (Nemergut et al. 2013; Urban and Skelly 2006).

### 4.1 Historical Contingency and Priority Effects as Greater Late‐Stage Drivers

The persistent and increasingly greater role of historical contingency observed in this study highlights the importance of priority effects in freshwater microbial community assembly (Tables 1 and 2) (Chase 2007; Hawkes and Keitt 2015). Even when communities were exposed to identical environmental media and continuous immigration, those originating from different historical sources maintained distinct compositional trajectories (Figures 2 and 3). This pattern is consistent with the hypothesis that early‐arriving taxa can exert long‐lasting influences through priority effects, potentially by preempting resources or modifying the local environment that constrain the establishment success of later arrivals, ultimately leading to divergence among communities experiencing similar contemporary environments (Sprockett et al. 2018; Werner and Kiers 2015).

We emphasize that the dominance of historical contingency observed here does not exclude the continued operation of environmental selection or biotic interactions. Rather, historical dominance reflects the cumulative outcome of early priority effects operating within persistent environmental constraints, whereby initial colonists gain advantages that are maintained even as environmental filtering continues to act (Chase et al. 2009; Fukami 2015).

Once niche space becomes increasingly occupied, these early‐established advantages can effectively “lock in” community structure, reducing the capacity of subsequent dispersal or environmental variation to redirect community trajectories (Drake 1991; Fukami and Nakajima 2011). Such historical lock‐in provides a mechanistic explanation for why freshwater microbial communities may remain distinct long after initial colonization events (Rillig et al. 2015; Vass et al. 2020).

### 4.2 Temporal Dynamics of Relative Dispersal Limitation: From Early Assembly Windows to Dispersal Saturation

**FIGURE 4:**
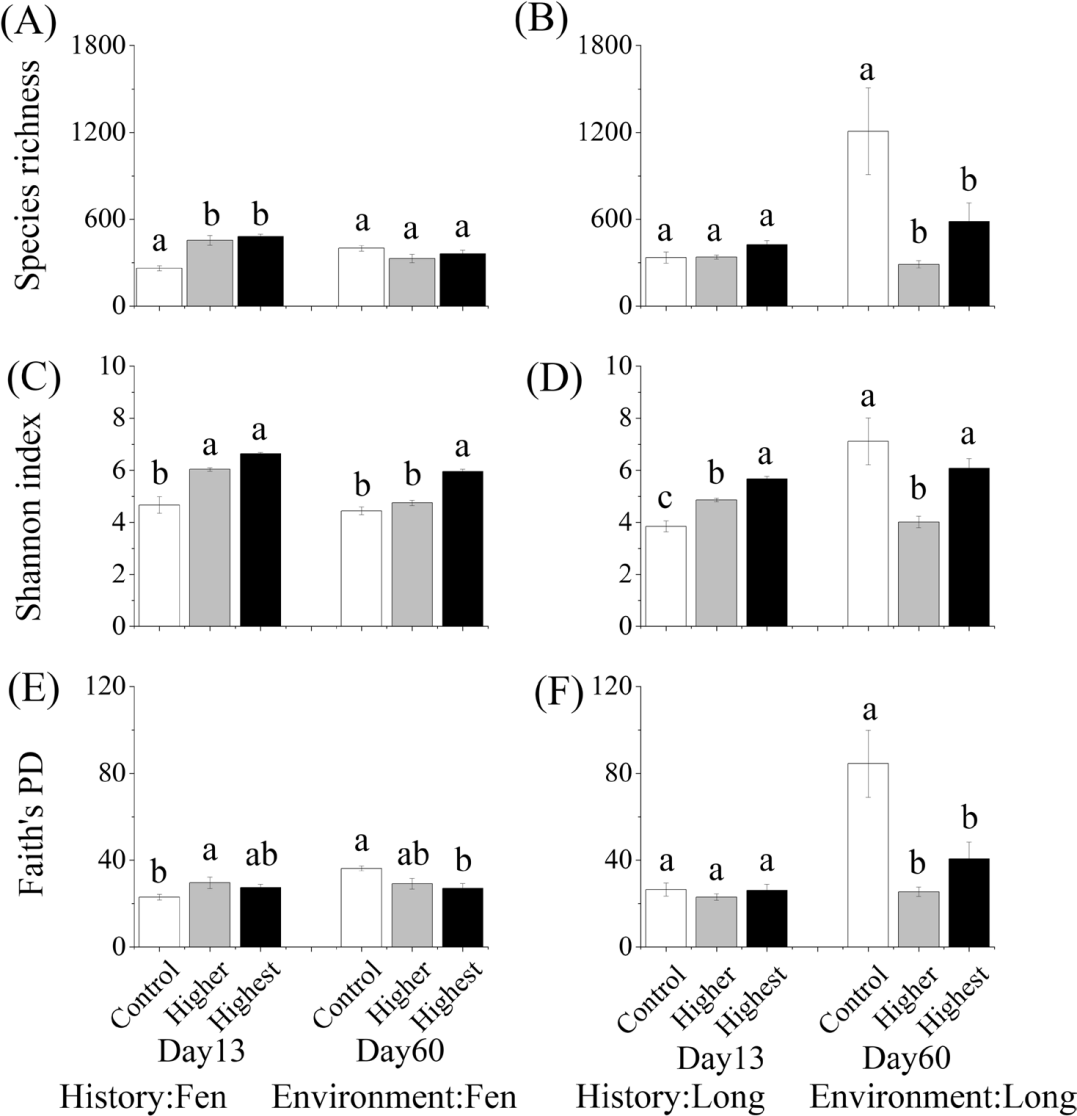
Alpha diversity metrics (ASV richness, Shannon index, Faith's PD) for Fen‐Fen and Long‐Long microcosms under three immigration treatments on day 13 and day 60. (A, C, E) The Fen‐Fen microcosms. (B, D, F) The Long‐Long microcosms. Differences were estimated using one‐way ANOVA, and means were compared by Tukey test. Data in (A–F) show mean ± SE (*n* = 8). In each panel, the same alphabet represents insignificant difference between the two treatments.

**FIGURE 5:**
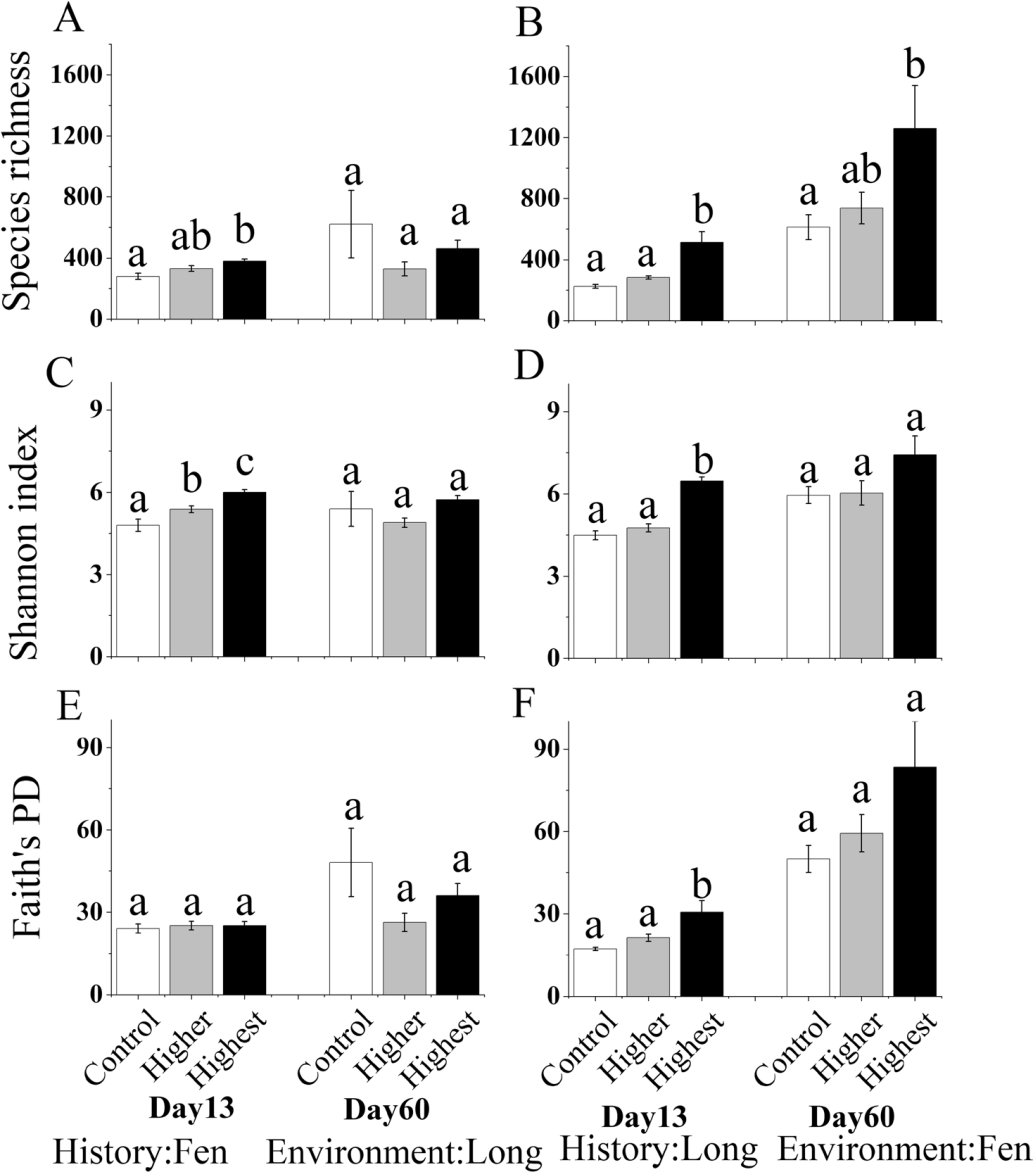
Alpha diversity metrics (ASV richness, Shannon index, Faith's PD) for Fen‐Fen and Long‐Long microcosms under three immigration treatments on day 13 and day 60. (A, C, E) The Fen‐Long microcosms. (B, D, F) The Long‐Fen microcosms.

Relative dispersal limitation exerted a strong influence during the early stage of community assembly (Figures 4 and 5), as evidenced by the positive effects of immigration on alpha diversity and community composition (Zhang et al. 2019). This pattern suggests that early assembly was characterized by an open colonization window, during which taxa arriving from the regional species pool were able to establish and contribute substantially to local community structure (Albright and Martiny 2018; De Meester et al. 2016). At this stage, available niche space and relatively weak biotic resistance likely facilitated successful immigration, consistent with predictions from metacommunity theory (Hubbell 2001; Leibold and Chase 2018).

In contrast, the influence of dispersal diminished markedly by the late stage of the experiment (Figures 4 and 5), indicating a transition toward dispersal saturation (Chase 2010). As communities matured, increasing niche occupation, competitive interactions, and established biotic networks likely reduced the establishment success of incoming taxa, even under elevated immigration (HilleRisLambers et al. 2012; Konopka 2009). This temporal shift underscores that dispersal limitation is not a static property of microbial communities but varies predictably across successional stages (Evans and Allison 2019; Matias et al. 2013). The two sampling time points were selected to represent early establishment (day 13) and a relatively stabilized late stage (day 60) based on prior microcosm studies and preliminary observations (Jiao et al. 2019; Zhang and Zhang 2015). While finer temporal resolution could reveal additional transient dynamics, the strong contrast between these stages provides clear evidence for a temporal shift in dominant assembly mechanisms (Jiao et al. 2020; Zhang et al. 2019).

It is important to note that our experimental design compared ambient levels of natural airborne dispersal against artificially enhanced immigration. Therefore, our findings regarding ‘dispersal limitation’ should be interpreted as the effect of increasing dispersal above background levels. The strong early‐stage effect we observed indicates that communities were indeed limited by the supply of immigrants relative to the ambient deposition, and that increasing this supply could overcome this limitation. This design reflects a realistic ecological scenario where some level of dispersal is almost always present.

### 4.3 The Role of Environmental Selection in Shaping, but Not Overriding, Assembly Trajectories

Environmental selection appeared to exert a consistent influence on community composition throughout the experiment (Tables 1 and 2; Figure S1), confirming its foundational role in freshwater microbial assembly (Graham et al. 2016; Liu et al. 2015). Differences in water chemistry between river and spring media filtered bacterial taxa and shaped phylogenetic and taxonomic patterns, demonstrating that environmental conditions constrain the pool of taxa capable of persisting locally (Liao et al. 2016; Nelson et al. 2016).

However, the relative explanatory power of environmental selection declined over time compared with historical contingency. This pattern suggests that environmental conditions act primarily as a template that defines the boundaries of viable community states, while historical processes determine which taxa ultimately dominate within those boundaries (Caruso et al. 2011; Karimi et al. 2017). Once priority effects have established stable configurations, environmental filtering alone may be insufficient to restructure communities without substantial disturbance (Bell 2010; Jiao et al. 2017). These findings highlight the need to move beyond binary frameworks contrasting deterministic and stochastic processes, toward a temporally explicit view in which environmental selection, dispersal, and history interact dynamically to shape community assembly (Gilbert et al. 2012; Stegen et al. 2013).

### 4.4 Implications, Generality, and Limitations

The strong path dependency revealed in this study has important implications for understanding and managing freshwater microbial communities. In natural systems, disturbances such as flooding, water diversion, or pollution can reset community history, creating new assembly windows during which dispersal and priority effects may strongly influence subsequent trajectories (Allison and Martiny 2008; Shade et al. 2012). Our results suggest that the identity of early colonists following such disturbances may have lasting consequences for community structure, even when environmental conditions later stabilize (Hawkes et al. 2017; Jurburg et al. 2017).

Although our conclusions are derived from a river–spring freshwater system, the proposed time‐dependent assembly framework is intended to be conceptually transferable rather than system‐specific. Similar successional shifts in the relative importance of dispersal, environment, and history are likely to occur in other freshwaters ecosystems experiencing episodic disturbance and dispersal, and thus can be empirically tested across systems (Logares et al. 2013; Wang et al. 2013).

Our experimental design, involving a 40% medium refreshment every 3 days, introduced a recurring physical disturbance. This washout event likely imposes a selective filter favoring fast‐growing or planktonic taxa capable of rapid recolonization and may have contributed to the observed decline in the influence of dispersal over time. While this is a common feature of semi‐continuous culture systems designed to maintain environmental conditions, it represents a deviation from completely static natural water bodies. This disturbance regime may have accelerated the successional process and potentially amplified the importance of priority effects, as early colonizers able to withstand this washout could more effectively preempt niche space. Future studies could employ flow‐through or chemostat systems with lower dilution rates to mitigate this effect.

A limitation of our study is that the ‘environmental selection’ treatment was a composite of multiple, co‐varying physicochemical factors (e.g., temperature, nutrients). While our primary aim was to quantify the role of environment as an integrated selective force, this design precludes us from identifying the specific variables driving community divergence. Future studies could employ more reductionist approaches, manipulating individual environmental parameters (e.g., temperature or a specific nutrient) within a common water background to pinpoint the causal agents of selection.

One potential limitation of this study is the use of high‐dose gamma irradiation (100 kGy) to sterilize water media, which may alter dissolved organic matter and nutrient availability. While we did not perform detailed chemical analyses following irradiation, both environmental media were subjected to identical sterilization procedures, ensuring that any chemical alterations were consistent across treatments.

Importantly, our experimental aim was to create controlled and reproducible environmental conditions rather than to fully replicate natural water chemistry. Therefore, the relative differences between environmental treatments remain interpretable within the context of this study. Nevertheless, future studies incorporating detailed chemical characterization of irradiated media would help further evaluate the potential influence of irradiation on microbial community assembly.

Another potential limitation of this study is the difference in initial community richness between the two source communities. Communities with higher initial richness may have a greater probability of maintaining diversity during assembly, which could potentially influence observed patterns. However, several lines of evidence suggest that initial richness alone does not explain the observed community divergence. First, sequencing depth was standardized across samples through rarefaction. Second, Bray–Curtis dissimilarity, which is less sensitive to richness differences, was used to quantify community composition. Third, PERMANOVA results consistently showed that historical effects remained significant across treatments and time points. Importantly, if initial richness were the dominant driver, communities would be expected to converge under shared environmental conditions over time. Instead, we observed persistent divergence among communities, consistent with strong historical contingency. Nevertheless, future studies incorporating null model approaches or richness‐controlled simulations would further help disentangle the influence of initial diversity from historical effects.

Our observed taxonomic shifts lead to specific hypotheses about functional consequences. For example, the higher relative abundance of Cyanobacteria in Fen‐River history communities suggests these systems might have a higher potential for primary production and autotrophic carbon cycling. In contrast, the greater proportion of Bacteroidota, a phylum known for degrading complex high‐molecular‐weight organic matter (e.g., proteins and polysaccharides), in Longci‐Spring history communities could imply different capacities for organic matter turnover. Future research could directly test these hypotheses by coupling manipulative history experiments with metagenomic or metatranscriptomic analyses to link community composition to functional gene potential and expression, or by directly measuring ecosystem processes (e.g., production rates, extracellular enzyme activities) to see if the divergent community trajectories we observed translate into divergent ecosystem functioning.

We acknowledge that 16S rRNA gene amplicon sequencing primarily captures taxonomic and phylogenetic patterns rather than functional activity. Accordingly, our inferences focus on community assembly processes inferred from compositional structure, rather than direct links to ecosystem functioning, which warrants future investigation using functional or activity‐based approaches (Louca et al. 2016; Zhang et al. 2025).

We also acknowledge that our comprehensive model, while highly significant, explained only a portion of the total community variation (e.g., ~50% at day 60). The substantial unexplained variance (residuals) highlights the inherent stochasticity in microbial community assembly, likely arising from ecological drift, unmeasured biotic interactions, and subtle variations in colonization history not captured by our treatments. Thus, while our results demonstrate strong path dependency and a greater role of history, community trajectories are not strictly deterministic or perfectly predictable. This interplay of predictable (history, environment) and stochastic (drift, chance) elements is a hallmark of ecological assembly.

## 5 Conclusion

In conclusion, this study demonstrates that freshwater microbial community assembly is governed by a dynamic and time‐dependent interplay among relative dispersal limitation, environmental selection, and historical contingency. Early in succession, community structure is strongly influenced by dispersal processes, reflecting an open assembly window during which immigration shapes diversity and composition. As succession proceeds, however, the influence of dispersal diminishes, while historical contingency persists and ultimately becomes a greater driver through priority effects that lock communities into distinct assembly trajectories. Environmental selection provides an important template throughout this process but is insufficient to outweigh established historical legacies once niche space becomes saturated. Together, these findings support a predictable assembly framework in which the relative importance of assembly mechanisms shifts over time, highlighting strong path dependency in freshwater microbial communities. This framework improves our ability to anticipate microbial community responses to disturbance and environmental change and has important implications for the management and restoration of freshwater ecosystems.

## Author Contributions

**Fenguo Zhang:** conceptualization (equal), formal analysis (equal), funding acquisition (equal), project administration (equal), software (equal), writing – original draft (equal). **Xiaoting Zhang:** data curation (equal), methodology (equal). **Dongqing Yan:** resources (equal), supervision (equal), visualization (equal). **Yufeng Jing:** conceptualization (equal), data curation (equal), investigation (equal), validation (equal). **Yongji Wang:** conceptualization (equal), funding acquisition (equal), project administration (equal), writing – review and editing (equal).

## Funding

This work was supported by the project for Local Science and Technology Development Guided by the Central Government in Shanxi Province, China (YDZJSX2025D060), and Research Project Supported by Shanxi Scholarship Council of China (2023‐110 and 2024‐089).

## Conflicts of Interest

The authors declare no conflicts of interest.

## Supporting information


**Figure S1:** The UPGMA clustering tree for each group at the phylum level. The Weighted Unifrac distance matrix was used for UPGMA cluster analysis, and the cluster results were integrated with the relative species abundance of each group at the phylum level.
**Table S1:** Information about the 10 sites to collect dust for the regional microbial pool.
**Table S2:** Physical and chemical properties of water samples.
**Table S3:** The bacteria community structure of dust mixture and initial non‐sterile water samples.
**Table S4:** Microcosms information.

## Data Availability

The raw data supporting the conclusions of this article will be made available by the authors, without undue reservation. The 16S rRNA gene sequence data were deposited in the NCBI Sequence Read Archive under accession number PRJNA985050.
