# Supplementary material for: Historical Contingency Drives Freshwater Microbial Community Assembly Across Successional Time
Source: Ecol Evol. 2026 Jun 9;16(6):e73819. doi: 10.1002/ece3.73819 (PMC13249528; doi:10.1002/ece3.73819)
Supplement: Supplementary file 1 — Figure S1: The UPGMA clustering tree for each group at the phylum level. The Weighted Unifrac distance matrix was used for UPGMA cluster analysis, and the cluster results were integrated with the relative species abundance of each group at the phylum level. Table S1: Information about the 10 sites to collect dust for the regional microbial pool. Table S2: Physical and chemical properties of water samples. Table S3: The bacteria community structure of dust mixture and initial non‐sterile water samples. Table S4: Microcosms information. [file ECE3-16-e73819-s001.docx]

**Historical contingency drives freshwater microbial community assembly across successional time**

**Fenguo Zhang^1^, Xiaoting Zhang^1^, Dongqing Yan^1^, Yufeng Jing^1^, Yongji Wang^1*^**

^1^College of Life Science, Shanxi Engineering Research Center of Microbial application technologies, Shanxi Normal University, Taiyuan, Shanxi, China

*** Correspondence:** Yongji Wang: wangyongji126@126.com

**Supplementary Figure**

**Supplementary Tables**

**Supplementary Fig.1** The UPGMA clustering tree for each group at the phylum level. The Weighted Unifrac distance matrix was used for UPGMA cluster analysis, and the cluster results were integrated with the relative species abundance of each group at the phylum level.


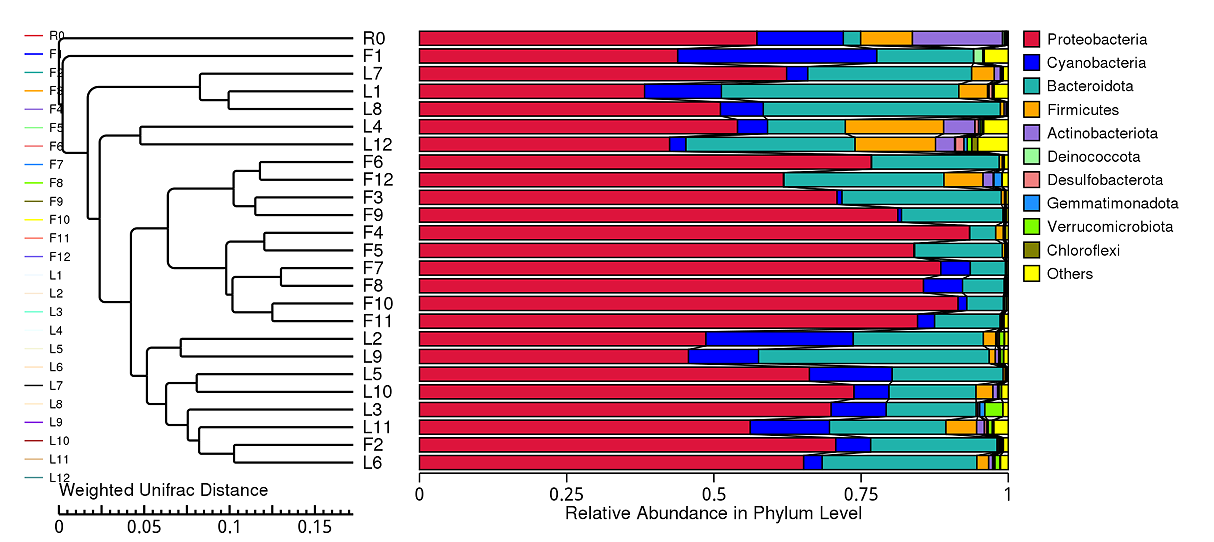


**Supplementary Table 1.** Information about the 10 sites to collect dust for the regional microbial pool

| **Site ID** | **Location** | **Altitude (m)** | **Habitat type** | **Description** |
| --- | --- | --- | --- | --- |
| A | 111°05′ E; 35°56′ N | 449 | Residential area | Dust collected from building walls in urban residential zone |
| B | 111°29′ E; 36°06′ N | 449 | Campus | Outdoor surfaces near university buildings |
| C | 111°29′ E; 36°01′ N | 447 | Campus underground | Dust from basement structures |
| D | 111°10′ E; 36°10′ N | 445 | Residential | Building surfaces with low human disturbance |
| E | 111°33′ E; 36°31′ N | 448 | Industrial | Dust from steel factory area |
| F | 111°20′ E; 36°04′ N | 450 | Campus | Academic building surroundings |
| G | 111°47′ E; 36°20′ N | 449 | Campus | Outdoor experimental facilities |
| H | 111°29′ E; 36°04′ N | 447 | Campus | Sports ground structures |
| I | 111°43′ E; 35°55′ N | 440 | Residential | Dormitory building surfaces |
| J | 111°15′ E; 35°58′ N | 450 | Greenhouse | Agricultural experimental greenhouse |

Note: Dust samples were collected from surfaces not recently disturbed to represent long-term airborne microbial deposition.

**Supplementary Table 2.** Physical and chemical properties of water samples

| **index** | **Longci Spring water** | **Fen River water** |
| --- | --- | --- |
| DO | 12.6 | 11.0 |
| AN | 0.040 | 1.050 |
| COD_Cr_ | 4 | 15 |
| TP | 0.18 | 0.16 |
| TN | 2.67 | 2.48 |
| pH | 7.34 | 7.22 |
| T | 20.0 | 29.0 |

Note: DO: Dissolved oxygen (mg/L); AN: Ammonia nitrogen (mg/L); COD_cr_: Chemical oxygen demand (mg/L); TP: Total phosphorus (mg/L); TN: Total nitrogen (mg/L); T: Temperature (℃)

**Supplementary Table 3.** The bacteria community structure of dust mixture and initial non-sterile water samples

| Sample | Observed-species | Shannon | Simpson | Chao1 | Goods-coverage |
| --- | --- | --- | --- | --- | --- |
| dust mixture | 488 | 6.066 | 0.939 | 492.690 | 1.000 |
| Longci Spring | 1489 | 6.506 | 0.945 | 1633.264 | 0.996 |
| Fen River | 843 | 5.877 | 0.904 | 937.759 | 0.997 |

**Supplementary Table 4.**

Microcosms information

| history | environment | immigration | Day13 | | Day 60 | |
| --- | --- | --- | --- | --- | --- | --- |
| (non-sterile water) | (sterile water) |  | sample | group | sample | group |
|  |  | regional air pool | R0 | R0 |  |  |
| Fen River | Fen River | Control | F1.1 | F1 | L1.1 | L1 |
|  |  |  | F1.2 | F1 | L1.2 | L1 |
|  |  |  | F1.3 | F1 | L1.3 | L1 |
|  |  |  | F1.4 | F1 | L1.4 | L1 |
|  |  |  | F1.5 | F1 | L1.5 | L1 |
|  |  |  | F1.6 | F1 | L1.6 | L1 |
|  |  |  | F1.7 | F1 | L1.7 | L1 |
|  |  |  | F1.8 | F1 | L1.8 | L1 |
|  |  | higher | F2.1 | F2 | L2.1 | L2 |
|  |  |  | F2.2 | F2 | L2.2 | L2 |
|  |  |  | F2.3 | F2 | L2.3 | L2 |
|  |  |  | F2.4 | F2 | L2.4 | L2 |
|  |  |  | F2.5 | F2 | L2.5 | L2 |
|  |  |  | F2.6 | F2 | L2.6 | L2 |
|  |  |  | F2.7 | F2 | L2.7 | L2 |
|  |  |  | F2.8 | F2 | L2.8 | L2 |
|  |  | highest | F3.1 | F3 | L3.1 | L3 |
|  |  |  | F3.2 | F3 | L3.2 | L3 |
|  |  |  | F3.3 | F3 | L3.3 | L3 |
|  |  |  | F3.4 | F3 | L3.4 | L3 |
|  |  |  | F3.5 | F3 | L3.5 | L3 |
|  |  |  | F3.6 | F3 | L3.6 | L3 |
|  |  |  | F3.7 | F3 | L3.7 | L3 |
|  |  |  | F3.8 | F3 | L3.8 | L3 |
| Longci | Longci | Control | F4.1 | F4 | L4.1 | L4 |
|  |  |  | F4.2 | F4 | L4.2 | L4 |
|  |  |  | F4.3 | F4 | L4.3 | L4 |
|  |  |  | F4.4 | F4 | L4.4 | L4 |
|  |  |  | F4.5 | F4 | L4.5 | L4 |
|  |  |  | F4.6 | F4 | L4.6 | L4 |
|  |  |  | F4.7 | F4 | L4.7 | L4 |
|  |  |  | F4.8 | F4 | L4.8 | L4 |
| history | environment | immigration | Day13 | | Day 60 | |
| (non-sterile water) | (sterile water) |  | sample | group | sample | group |
|  |  | Higher | F5.1 | F5 | L5.1 | L5 |
|  |  |  | F5.2 | F5 | L5.2 | L5 |
|  |  |  | F5.3 | F5 | L5.3 | L5 |
|  |  |  | F5.4 | F5 | L5.4 | L5 |
|  |  |  | F5.5 | F5 | L5.5 | L5 |
|  |  |  | F5.6 | F5 | L5.6 | L5 |
|  |  |  | F5.7 | F5 | L5.7 | L5 |
|  |  |  | F5.8 | F5 | L5.8 | L5 |
|  |  | Highest | F6.1 | F6 | L6.1 | L6 |
|  |  |  | F6.2 | F6 | L6.2 | L6 |
|  |  |  | F6.3 | F6 | L6.3 | L6 |
|  |  |  | F6.4 | F6 | L6.4 | L6 |
|  |  |  | F6.5 | F6 | L6.5 | L6 |
|  |  |  | F6.6 | F6 | L6.6 | L6 |
|  |  |  | F6.7 | F6 | L6.7 | L6 |
|  |  |  | F6.8 | F6 | L6.8 | L6 |
| Fen River | Longci | Control | F7.1 | F7 | L7.1 | L7 |
|  |  |  | F7.2 | F7 | L7.2 | L7 |
|  |  |  | F7.3 | F7 | L7.3 | L7 |
|  |  |  | F7.4 | F7 | L7.4 | L7 |
|  |  |  | F7.5 | F7 | L7.5 | L7 |
|  |  |  | F7.6 | F7 | L7.6 | L7 |
|  |  |  | F7.7 | F7 | L7.7 | L7 |
|  |  |  | F7.8 | F7 | L7.8 | L7 |
|  |  | Higher | F8.1 | F8 | L8.1 | L8 |
|  |  |  | F8.2 | F8 | L8.2 | L8 |
|  |  |  | F8.3 | F8 | L8.3 | L8 |
|  |  |  | F8.4 | F8 | L8.4 | L8 |
|  |  |  | F8.5 | F8 | L8.5 | L8 |
|  |  |  | F8.6 | F8 | L8.6 | L8 |
|  |  |  | F8.7 | F8 | L8.7 | L8 |
|  |  |  | F8.8 | F8 | L8.8 | L8 |
|  |  | Highest | F9.1 | F9 | L9.1 | L9 |
|  |  |  | F9.2 | F9 | L9.2 | L9 |
|  |  |  | F9.3 | F9 | L9.3 | L9 |
| history | environment | immigration | Day13 | | Day 60 | |
| (non-sterile water) | (sterile water) |  | sample | group | sample | group |
|  |  |  | F9.4 | F9 | L9.4 | L9 |
|  |  |  | F9.5 | F9 | L9.5 | L9 |
|  |  |  | F9.6 | F9 | L9.6 | L9 |
|  |  |  | F9.7 | F9 | L9.7 | L9 |
|  |  |  | F9.8 | F9 | L9.8 | L9 |
| Longci | Fen River | Control | F10.1 | F10 | L10.1 | L10 |
|  |  |  | F10.2 | F10 | L10.2 | L10 |
|  |  |  | F10.3 | F10 | L10.3 | L10 |
|  |  |  | F10.4 | F10 | L10.4 | L10 |
|  |  |  | F10.5 | F10 | L10.5 | L10 |
|  |  |  | F10.6 | F10 | L10.6 | L10 |
|  |  |  | F10.7 | F10 | L10.7 | L10 |
|  |  |  | F10.8 | F10 | L10.8 | L10 |
|  |  | Higher | F11.1 | F11 | L11.1 | L11 |
|  |  |  | F11.2 | F11 | L11.2 | L11 |
|  |  |  | F11.3 | F11 | L11.3 | L11 |
|  |  |  | F11.4 | F11 | L11.4 | L11 |
|  |  |  | F11.5 | F11 | L11.5 | L11 |
|  |  |  | F11.6 | F11 | L11.6 | L11 |
|  |  |  | F11.7 | F11 | L11.7 | L11 |
|  |  |  | F11.8 | F11 | L11.8 | L11 |
|  |  | Highest | F12.1 | F12 | L12.1 | L12 |
|  |  |  | F12.2 | F12 | L12.2 | L12 |
|  |  |  | F12.3 | F12 | L12.3 | L12 |
|  |  |  | F12.4 | F12 | L12.4 | L12 |
|  |  |  | F12.5 | F12 | L12.5 | L12 |
|  |  |  | F12.6 | F12 | L12.6 | L12 |
|  |  |  | F12.7 | F12 | L12.7 | L12 |
|  |  |  | F12.8 | F12 | L12.8 | L12 |
